# A Survey of Blind Modulation Classification Techniques for OFDM Signals

**DOI:** 10.3390/s22031020

**Published:** 2022-01-28

**Authors:** Anand Kumar, Sudhan Majhi, Guan Gui, Hsiao-Chun Wu, Chau Yuen

**Affiliations:** 1 Department of Electrical Engineering, Indian Institute of Technology Patna, Patna 801103, India; anand_1921ee15@iitp.ac.in; 2 Department of Electrical Communication Engineering, Indian Institute of Science (IISc), Bangalore 560012, India; 3 College of Telecommunications and Information Engineering, Nanjing University of Posts and Telecommunications, Nanjing 210003, China; guiguan@njupt.edu.cn; 4 School of Electrical Engineering and Computer Science, Louisiana State University, Baton Rouge, LA 70803, USA; wu@ece.lsu.edu; 5 Engineering Product Development (EPD) Pillar, Singapore University of Technology and Design, Singapore 487372, Singapore; yuenchau@sutd.edu.sg

**Keywords:** blind modulation classification, orthogonal frequency division multiplexing, higher-order cumulant and cyclic cumulant, maximum-likelihood, maximum a posteriori, deep learning, convolutional neural networks, probability of correct classification, testbed implementation

## Abstract

Blind modulation classification (MC) is an integral part of designing an adaptive or intelligent transceiver for future wireless communications. Blind MC has several applications in the adaptive and automated systems of sixth generation (6G) communications to improve spectral efficiency and power efficiency, and reduce latency. It will become a integral part of intelligent software-defined radios (SDR) for future communication. In this paper, we provide various MC techniques for orthogonal frequency division multiplexing (OFDM) signals in a systematic way. We focus on the most widely used statistical and machine learning (ML) models and emphasize their advantages and limitations. The statistical-based blind MC includes likelihood-based (LB), maximum a posteriori (MAP) and feature-based methods (FB). The ML-based automated MC includes k-nearest neighbors (KNN), support vector machine (SVM), decision trees (DTs), convolutional neural networks (CNNs), recurrent neural networks (RNNs), and long short-term memory (LSTM) based MC methods. This survey will help the reader to understand the main characteristics of each technique, their advantages and disadvantages. We have also simulated some primary methods, i.e., statistical- and ML-based algorithms, under various constraints, which allows a fair comparison among different methodologies. The overall system performance in terms bit error rate (BER) in the presence of MC is also provided. We also provide a survey of some practical experiment works carried out through National Instrument hardware over an indoor propagation environment. In the end, open problems and possible directions for blind MC research are briefly discussed.

## 1. Introduction

Blind modulation classification (MC) determines the modulation type of the received signal, ensuring proper demodulation and retrieval of the transmitted data [1,2,3,4]. Recently, MC has played a significant role in both military and civilian communications, such as cognitive radio, signal intelligence, link adaptation, signal control, and SDR [3,4,5,6,7]. With an intelligent receiver, blind parameter estimation and classification algorithms may be used, resulting in a substantial increase in spectral efficiency since no predefined training or pilot sequence is needed [8,9,10].

Over the years, various MC algorithms for single-carrier (SC) systems have been developed, which can be divided into likelihood-based (LB) and feature-based methods (FB) [1,5,6,11,12,13,14,15,16,17]. Although the LB approaches are optimal in a Bayesian context, they have high computational complexity [11]. They often necessitate prior information about the signal parameters in order to distinguish modulation formats, which is typically undesirable in an intelligent or adaptive transceiver system. Furthermore, FB algorithms, which consist of features extraction and classifier construction, usually provide a sub-optimal solution. They are inherently simpler to implement, have less computational complexity, and may not necessitate prior information about the signal parameters and channel statistics. To identify the modulation schemes, existing FB approaches extract specific features, such as cumulants [12,13], cyclic statistics [5,6,14,15,16], and wavelet transform [17], and use threshold values to distinguish the extracted features. As a result, they are better fit for fading and additive white Gaussian noise (AWGN) channels. The algorithms [5,6,14,15,16,18,19,20,21] based on higher-order cyclic statistics are reliable and perform well in flat fading as well as in frequency-selective fading channels. They consider M-ary phase-shift keying (M-PSK) and M-ary quadrature amplitude modulation (M-QAM) modulation schemes by using non-zero cyclic frequencies of received signals. The combination of higher-order correlation-, cumulant-, cyclic cumulant-, and cyclostationarity-based MC algorithm for multiple-antenna systems is analyzed in [5]. The algorithm described in [6] is designed for single-antenna and single-carrier (SC) systems. It requires the combined features of cumulants and cyclic cumulants and performs well over flat fading channels. Furthermore, the algorithm proposed in [5,6] can also distinguish various quadrature PSK (QPSK) variants, such as offset QPSK (OQPSK), minimum-shift keying (MSK), and π/4-QPSK modulation types.

Recent advances in machine learning (ML) and data science have resulted in its extensive application in various fields. Artificial intelligence (AI) and other advanced ML approaches have significantly improved state-of-the-art outcomes in computer vision, speech recognition [22], drug discovery, genomics, and, most recently, physical layer communication [23]. MC algorithms [24,25,26,27,28,29,30,31,32,33,34,35,36] focused on various ML algorithms. In [24], the MC technique is evaluated using genetic programming (GP) and K-nearest neighbor (KNN). Cumulants are utilized by GP as input features to distinguish modulation types. In [25], extreme learning machine (ELM) and higher-order statistics-based MC algorithms for multiple antenna systems are presented. Convolutional neural networks (CNNs) are explored in [26] that can distinguish modulation schemes even at low signal-to-noise ratio (SNR) scenarios. Furthermore, CNN-based MC techniques are robust to prediction errors on carrier phase offset and SNR. The approach investigated in [27] extracts unique characteristics using higher-order cumulants (HOCs), and then the feed-forward neural network model is developed to distinguish modulation schemes. In comparison to typical centralized training, distributed learning-based MC (DistMC) based on several edge devices can achieve a faster training process and reduce communication costs through collaborative training [28]. Multi-task learning (MTL) based MC has a single trained model for all SNRs under carrier frequency offset (CFO) and phase offset (PO) conditions [29,30]. In [35], an adversarial transfer learning-based MC developed a framework for SC systems that combines transfer learning with adversarial networks to handle the problem of limited data in a realistic scenario. A complex-valued network [36] is presented to illustrate the enormous potential for MC and show the higher classification performance as compared to the real-valued network. The authors [37] studied a phoneme-based distribution regularization algorithm for speech enhancement by utilizing speech recognition information in the modulation domain. However, the approaches mentioned above [1,5,6,11,12,13,14,15,16,17,24,25,26,27,28,29,30,31,32,33,34,35,36] are only applicable to SC systems.

Orthogonal frequency division multiplexing (OFDM) is a well-known multicarrier modulation technology used in advanced wireless communications systems. OFDM is employed in the 4G Third-Generation Partnership Project (3GPP) Long Term Evolution-Advanced (LTE/LTE-A), Worldwide Interoperability for Microwave Access (WiMAX), and high-speed wireless local area network (WLAN) standards such as 802.11n [19]. It is also an integral part of 5G New Radio (NR) cellular. The key feature of OFDM is the ability to convert frequency-selective fading to flat fading channels. Due to the high spectrum utilization and strong anti-multipath interference ability, the OFDM modulation scheme has been employed as the main transmission approach for high data rate systems [38,39]. M-PSK and M-QAM are the two most popular modulation schemes that are used with OFDM. MC for OFDM signals is a critical research challenge for 5G and beyond wireless communication, where AI would be a fundamental aspect of the communication system [40,41,42,43].

Various MC algorithms for the OFDM systems were carried out in [44,45,46,47,48,49,50,51,52,53,54,55,56,57,58,59,60,61,62,63,64,65,66,67,68,69,70,71,72,73,74,75,76,77,78,79,80,81,82,83,84,85,86,87,88,89,90,91,92,93,94,95,96,97]. The algorithms for multiple-input multiple-output and OFDM (MIMO-OFDM) systems based on deep neural network (DNN) and Gibbs sampling are investigated in [44]. Moreover, these methods are restricted to known channel conditions and/or perfect synchronization. The likelihood-based MC algorithm for index modulation investigated in [47,71] is applicable to both known and unknown channel state information (CSI). However, both techniques require perfect synchronization classification of M-PSK/M-QAM modulation types. The likelihood and maximum a posteriori [50] based MC approach are employed when CSI is known. The MC approach based on the statistical features of the received OFDM signal is studied in [61]. This technique uses mean, skewness, and kurtosis as features to distinguish QPSK, 16-QAM, and 64-QAM modulation schemes. However, this technique does not perform well with timing and frequency synchronization errors. The MC algorithm based on amplitude moments is discussed in [62]. This method distinguishes between 16-QAM and 64-QAM modulation schemes by using the correlation between any two subcarriers. The non-parametric Kolmogorov–Smirnov (KS) based technique presented in [98,99] is used to classify M-PSK/M-QAM modulation schemes. It operates in the presence of known timing offset and unknown frequency and phase offsets, and the non-Gaussian noise channel. Most of the above MC algorithms for the OFDM signal are restricted to known CSI and/or perfect synchronization cases. Moreover, a discrete Fourier transform (DFT) and normalized higher-order cumulant [63] based blind MC is discussed to classify the lower-order digital modulation schemes for the OFDM system. However, the classification accuracy is unsatisfactory, subjected to channel degradation. In [96], the authors developed a high-performance deep residual network (ResNet) with a triple-skip residual stack (TRNN) based MC algorithm for real-time OFDM signal classification in dynamic fading channel conditions.

The objective of this paper is to present a comprehensive review of various MC techniques for OFDM signals. The statistical approach and the AI approach are two main classes of MC algorithms that will be discussed in detail. We concentrate on the most common statistical and ML models, emphasizing their benefits and drawbacks. The contributions of various research papers are summarized into compact forms. This will make it easier for the reader to recognize the important features of each approach. Furthermore, we also present results obtained by applying some statistical and ML algorithms with a testbed based on the National Instrument (NI) radio frequency (RF) hardware over an indoor transmission environment. Finally, challenges and potential research directions are briefly explored.

**Figure 1:**
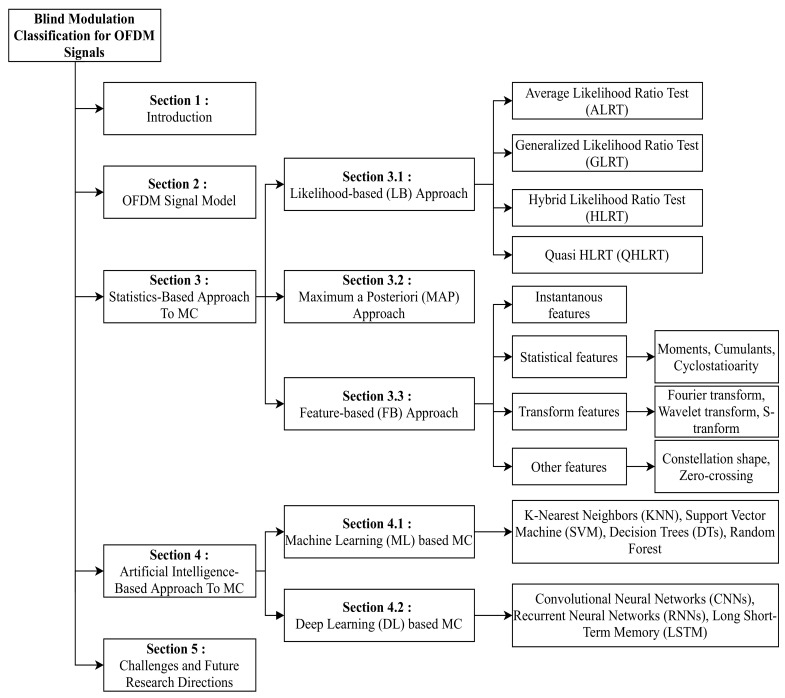
The organization of the paper.

The remainder of the paper is organized as follows. The signal model of the received OFDM system is presented in Section 2. The statistical approach for MC is discussed in Section 3. We summarize the advantages and the limitations of AI models in MC in Section 4. Finally, challenges and future research directions involved in MC are discussed in Section 5. The organization of the paper is provided in Figure 1. The abbreviation used in the rest of the paper is listed in Table 1.

## 2. OFDM Signal Model

**Figure 2:**
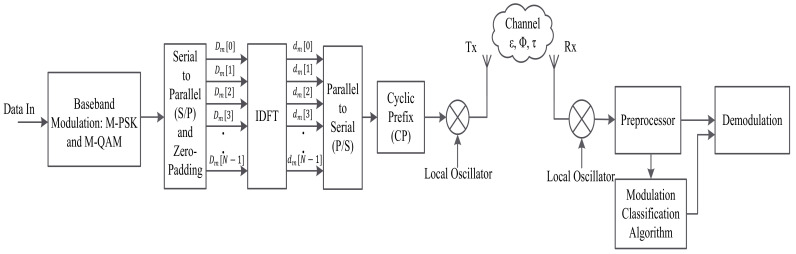
Block diagram of blind modulation classification for OFDM system.

The system model of MC for the OFDM system is shown in Figure 2. It consists of an adaptive OFDM transmitter, a receiver with statistics-based MC, ML-based MC, and DL-based MC. The transmitter can adjust its baseband modulation format and the number of subcarriers according to the requirement of the data rate and the available CSI. The signal is transmitted over a frequency-selective fading channel. This channel introduces all kinds of impairments into the transmitted signal, including timing, frequency, and phase offsets. The receiver consists of an MC system pre-processing block and selection of a proper MC algorithm. In the following subsections, we provide the mathematical framework of the OFDM signal for MC.

The discrete baseband OFDM samples $d_{m}\left[n\right]$ of the *m*th OFDM symbol, obtained by *N*-point inverse discrete Fourier transform (IDFT), which can be written as

(1) $$d_{m}\left[n\right]=\sum_{k=0}^{N-1}D_{m}\left[k\right]e^{j2\pi kn/N},\;0\leq n\leq N-1,$$

where $N=\rho_{s}\times N_{d}$, $\rho_{s}$ is the oversampling factor, $N_{d}$ is the number of data subcarriers, and $D_{m}\left[k\right]$ is the baseband modulated oversampled data obtained by zero-padding the baseband modulated information, i.e., M-PSK/M-QAM and denoted by ${\hat{D}}_{m}\left[k\right]$. Thus, $D_{m}\left[k\right]$ is given by

(2) $$\begin{array}{c} D_{m}\left[k\right]=\left\{\begin{array}{cc} {\hat{D}}_{m}\left[k\right]\; & 0\leq k\leq N_{d}/2-1\\ {\hat{Z}}_{0}\; & N_{d}/2\leq k\leq N_{d}\left(\rho_{s}-1/2\right)-1\\ {\hat{D}}_{m}\left[k\right]\; & N_{d}\left(\rho_{s}-1/2\right)\leq k\leq N-1, \end{array}\right. \end{array}$$

where ${\hat{Z}}_{0}$ is a vector of zeros of length $N_{d}\left(\rho_{s}-1\right)$. To combat the effect of intersymbol interference (ISI), a cyclic-prefix (CP) of $N_{cp}$ samples from the end of the OFDM symbol are added at the beginning of the OFDM symbol before the transmission. The transmitted baseband OFDM symbol ${\bar{d}}_{m}\left[n\right]$ of length $N+N_{cp}$, with CP is then given by

(3) $$\begin{array}{c} {\bar{d}}_{m}\left[n\right]=\left\{\begin{array}{cc} d_{m}\left[n+N\right]\; & -N_{cp}\leq n\leq -1\\ d_{m}\left[n\right]\; & 0\leq n\leq N-1. \end{array}\right. \end{array}$$

After passing through a frequency-selective fading channel with impulse response g[l] of length *L*, the received baseband OFDM samples of the *m*th OFDM symbol are given by

(4) $$x_{m}\left[n\right]=e^{\left(j2\pi \epsilon n/N+\phi \right)}\sum_{l=0}^{L-1}g\left[l\right]{\bar{d}}_{m}\left[n-l-\tau \right]+\omega \left[n\right],\;0\leq n\leq N_{s}-1$$

where ϵ is the normalized carrier frequency offset (CFO), ϕ is the phase offset, τ is the symbol timing offset (STO), $N_{s}$ length of the OFDM symbol with CP, $N_{s}=N+N_{cp}$ and $N_{cp}\geq L$, and ω[n] is the AWGN with zero mean and variance $\sigma_{\omega }^{2}$.

## 3. Statistics-Based Approach to MC

### 3.1. LB Approach

In the LB system, MC refers to numerous composite hypothesis problems. The LB-MC is based on the assumption that the probability density function (PDF) of the analyzed waveform includes all classification information, depending on the embedded modulated signal. The average likelihood ratio test (ALRT) [47], generalized likelihood ratio test (GLRT) [46], and hybrid likelihood ratio test (HLRT) [47] are the main three LB-MC techniques studied in the literature, based on the model selected for the unknown parameter. In some works in the literature, quasi-ALRT [46] and quasi-HLRT [45,46] are also described.

ALRT: In this method, unknown parameters are considered random variables with specific PDFs. For the hypothesis $H_{j}$, which represents the *j*th modulation, j=1,2,…,M, the likelihood function (LF) is as follows

(5) $$\Lambda_{ALRT}^{j}=\sum_{v_{j}}\Lambda \left[x_{m}\left[n\right]|v_{j},H_{j}\right]p\left(v_{j}|H_{j}\right),$$

where $\Lambda [x_{m}\left[n\right]|v_{j},H_{j}]$ denotes the conditional LF of the received signal $x_{m}\left[n\right]$ associated with noise over $H_{j}$, conditioned on the undefined vector $v_{j}$ under $H_{j}$. By integrating over $v_{j}$ and using its known PDF, the problem is reduced to a basic hypothesis-testing problem. The conditional LF for a baseband complex AWGN is provided by

(6) $$\begin{array}{c} \Lambda \left[x_{m}\left[n\right]|v_{j},H_{j}\right]=\frac{1}{\pi N_{0}}exp\left(-\frac{\frac{1}{\eta_{0}}\sum_{n=0}^{N-1}{\left|x_{m}\left(n\right)-s_{m}\left[n\right]\right|}^{2}}{N_{0}}\right) \end{array}$$

where $N_{0}$ represents the power spectral density (PSD) of AWGN in W/Hz, with the auto-correlation $E\left\{\omega \left[n\right],\omega^{*}\left[n+\tau \right]\right\}=N_{0}\delta \left[n\right]$, with $E\left\{.\right\}$ denoting the expectation and * representing the complex conjugate. Furthermore, here $s_{m}\left[n\right]=e^{\left(j2\pi \epsilon n/N+\phi \right)}\sum_{l=0}^{L-1}g\left[l\right]{\bar{d}}_{m}\left[n-l-\tau \right]$. ALRT produces an optimal classifier in the Bayesian context when the chosen $p(v_{j}|H_{j})$ is the same as the true PDF.

GLRT: This approach considers the unknown parameters to be unknown deterministic. The best result is obtained by carrying out the so-called uniformly most powerful (UMP) test [100]. If UMP test does not exist or is difficult to obtain, a rational technique is used to estimate the unknown parameters based on the assumption that $H_{j}$ is true, and then utilize these estimations in ALRT as if they were accurate. When maximum likelihood is applied for estimations, the hypothesis test is known as GLRT. The unknown parameters of GLRT are, of course, considered deterministic unknowns, and LF under $H_{j}$ is provided by

(7) $$\Lambda_{GLRT}^{j}\left[x_{m}\left[n\right]\right]=\max_{v_{j}}\Lambda \left[x_{m}\left[n\right]|v_{j},H_{j}\right].$$

HLRT: This is the combined approaches of the above techniques, where the LF under $H_{j}$ is defined by

(8) $$\Lambda_{HLRT}^{j}=\max_{v_{j_{1}}}\sum_{v_{j_{2}}}\Lambda \left[x_{m}\left[n\right]|v_{j_{1}},v_{j_{2}},H_{j}\right]p\left(v_{j_{2}}|H_{j}\right)dv_{j_{2}},$$

where $v_{j}={\left[v_{j_{1}}^{†}v_{j_{2}}^{†}\right]}^{†}$ with † as the transpose and $v_{j_{1}}$ and $v_{j_{2}}$ are vectors of unknown parameters treated as unknown deterministic and random variables, respectively. Generally, $v_{j_{1}}$ and $v_{j_{2}}$ are made up of parameters and data symbols, respectively.

It is to be noted that ALRT necessitates multidimensional convergence, while GLRT necessitates multidimensional maximization. ALRT could be unrealistic due to the difficulties of performing multidimensional integration in the presence of a large number of unknown parameters and the requirement to know the PDFs. Furthermore, maximization over unknown parameters in GLRT results yield the same LF value for nested signal constellations, such as BPSK and QPSK, 16-QAM, and 64-QAM, resulting in inaccurate classification. However, with HLRT, averaging over unknown data symbols eliminates the GLRT problem of nested constellations. In the case of a two-hypothesis classification problem, a decision is made on the basis of

(9) $$\begin{array}{c} \Lambda_{H}^{\left(1\right)}\left[x_{m}\left[n\right]\right]/\Lambda_{H}^{\left(2\right)}\left[x_{m}\left[n\right]\right]\underset{H_{2}}{\overset{H_{1}}{\underset{<}{\geq }}}\eta_{l},l=A\left(\mathrm{ALRT}\right),G\left(\mathrm{GLRT}\right),H\left(\mathrm{HLRT}\right), \end{array}$$

where $\eta_{l}$ represents the threshold. The left-hand side of (9) represents the likelihood ratio, and the test is referred to as the ALRT, GLRT, and the HLRT, respectively, depending on the approach used to estimate the LF. The extension of (8) to multiple classes is simple. Likewise, the log function can be extended to the two members of the inequality (9). Table 2 lists multiple LB-MC algorithms proposed in the literature, outlining the modulation types, uncertain parameters, and the channel employed.

An LB-MC for the OFDM system is studied in [45]. The aim of this work is limited to reliable blind MC schemes. A maximum likelihood that provides optimal performance in the presence of AWGN is introduced. A sub-optimal classifier is obtained based on the optimal maximum-likelihood classifier to minimize the computational complexity. The accuracy of such classifiers is evaluated through Monte Carlo simulations. In the simulation, an OFDM system with 64 subcarriers is considered. The subcarriers are divided into 4 bands, each of which has 16 subcarriers. In each sub-band, four distinct modulation formats, namely BPSK, QPSK, 16-QAM, and 64-QAM, are used to transmit the signal according to the channel conditions. Perfect CSI is considered for the simulation. It is observed that the proposed sub-optimal algorithm achieves near to optimal performance with significantly less complexity. As a result, it can be used rather than signaling in realistic systems to improve spectral efficiency.

In the proposed method [46], a modified quasi-log-likelihood ratio (QLLR) based MC for the OFDM system is studied. The ALRT- and GLRT-based classifiers need few symbols to achieve acceptable classification performance in the presence of appropriate channel estimation with relatively high SNR. To achieve acceptable performance, a modified QLLR-based classifier needs high SNR and more symbols but their computational complexity remains lower compared to the ALRT- and GLRT-based classifiers. In order to classify QPSK, 16-QAM, and 64-QAM, the modified QLLR test is applied on received symbol sets. This method seems to be feasible if the operating point of SNR is comparatively high as compared to ALRT- and GLRT-based classifiers.

Another LB-MC for OFDM with index modulation (OFDM-IM) is analyzed in [47]. The modulation parameters in OFDM-IM often include the number of active subcarriers in addition to the constellation of signals, which distinguishes them from traditional modulations. Specifically, two MC cases are assumed. One is the MC with known CSI, and another is the MC with unknown CSI. ALRT, HLRT-LLR, and HLRT-energy-based classifiers are considered for the case of known CSI. When compared to ALRT, both HLRT-LLR and HLRT-energy have lower computational complexity, but show degradation in classification performance. In the case of unknown CSI, the energy-based detector is first used to recognize the active subcarriers, then the expectation-maximization (EM) algorithm is employed to estimate the CSI for each hypothesis. The number of subcarrier N=128, CP length $N_{cp}=15$, number of channel tap L=5 and Rayleigh channel are considered the simulation parameters. The simulation results revealed that with an increment in the observed data, the classification accuracy of MC with unknown CSI is near the MC with known CSI. Furthermore, a numerical analysis of MC for OFDM and OFDM-IM shows that OFDM-IM has less classification accuracy than the OFDM. It illustrates that OFDM-IM would have less MC efficiency than OFDM because of the identification of the additional parameter, i.e., the number of active subcarriers that would be necessary for OFDM-IM.

In [48], a MC for OFDM signal underwater acoustic multipath channel is studied. It works in the presence of unknown channel impulse response (CIR) and noise power. Channel is first estimated by the EM block. If the number of blocks in EM increases, the channel estimation increases accordingly. Then, the QHLRT method is used to classify the subcarrier modulations. The EM-block-QHLRT method is compared with the EM-QHLRT. The number of subcarrier N=1024, CP length $N_{cp}=N/4$, sampling frequency 48 kHz and acoustic Rayleigh channel are considered the simulation parameters for this technique. It is observed that after 5 dB SNR, the classification rate achieved by EM-block-QHLRT is higher than 90%, which shows a higher accuracy compared to the EM-QHLRT-based classifier.

In [49], an iterative EM-based MC algorithm is used for OFDM-SDR systems. The soft information provided by the channel decoder of bit-interleaved coded modulation iterative decoding (BICM-ID) scheme is utilized as a priori information to the proposed classifier. Simulation is done for the perfect CSI and imperfect CSI for higher-order modulations over the Rayleigh fading channel. The results show a slight difference between the perfect CSI and imperfect CSI, which shows the robustness of the suggested method. The suggested method improves significantly with iterations and outperforms traditional uncoded algorithms. The suggested method obtained acceptable classification performance in the presence of synchronization error, i.e., timing, frequency, and phase offset with reduced processing time. Furthermore, as the constellation size increases, the identification performance degrades. This is because of the less reliable soft information provided by the channel decoder.

### 3.2. Maximum a Posteriori (MAP) Approach

MC is the process of determining the modulation format of received signals from a set of *L* modulation formats $M=\left\{M_{j},\;j=1,2,\ldots ,M\right\}$, based on a series of *N* received samples $x_{m}=\left[x_{m}\left[0\right],x_{m}\left[1\right],\ldots ,x_{m}\left[N_{s}-1\right]\right]$. The maximum a posteriori (MAP) criteria can be used to find the optimal modulation classifier by using the Bayes decision principle [57]. For received signal $x_{m}$, the a posteriori probability of $M_{j}$ is defined as $P(M_{j}|x_{m})$, and the decision is made by

(10) $$\hat{M_{j}}=\arg\max_{M_{j}\in M}P\left(M_{j}|x_{m}\right),$$

Another well-known classifier originating from the MAP criteria is the ML classifier. The a posteriori probability can be expressed using the Bayes’ rule as

(11) $$P\left(M_{j}|x_{m}\right)=\frac{P\left(x_{m}|M_{j}\right)P\left(M_{j}\right)}{P(x_{m})},$$

where $P(x_{m}|M_{j})$ denotes the likelihood of the received samples $x_{m}$ when the modulation format $M_{j}$ is given, $P(M_{j})$ is the prior likelihood of the modulation format $M_{j}$, and $P(x_{m})$ is the marginal likelihood of the received samples $x_{m}$, which is independent of $M_{j}$. When all the candidate modulation formats are equiprobable, then the MAP classifier is identical to the ML classifier [51].

(12) $$\hat{M_{j}}=\arg\max_{M_{j}\in M}P\left(x_{m}|M_{j}\right).$$

Table 3 lists multiple MAP-based MC algorithms studied in the literature, outlining the modulation types, uncertain parameters, and the channel employed.

A MAP-based MC algorithm in time division duplex (TDD) based OFDM systems with adaptive QAM modulation is studied in [50]. It takes advantage of the channel reciprocity in TDD systems and the data rate of transmission. Unlike the signaling-free adaptive modulation technique, MC and data detection are decoupled here, resulting in significantly decreased computational complexity. Moreover, this technique utilizes the fixed bit allocation table (BAT) for all transmission frames. As a result, more symbols of the same modulation scheme can be employed to make a decision. Compared to the traditional ML method, simulations have validated the superior classification performance of the modified MAP algorithm. This technique allows adaptive modulation to be applied in wireless OFDM systems without reducing the effective data rate due to the signaling of the BAT.

A novel efficient MC algorithm in wireless TDD-based OFDM systems with adaptive modulation is analyzed in [51]. The frequency-selective behavior of the channel is experienced by a finite impulse response (FIR) filter model with Rayleigh fading coefficients. Jakes’ spectrum with the Doppler frequency $f_{dm}$ is used to model the time correlation of the different path coefficients. This adaptive modulation approach adapts modulation formats among BPSK, QPSK, 16-QAM, and 64-QAM to a group of two adjacent subcarriers. The conventional maximum-likelihood method is modified to a MAP classifier that uses reciprocity of the channels in TDD systems. Moreover, a less computationally complex classifier based on the MAP criteria is developed and evaluated, which is desirable for real-time implementations. The feasibility of complexity reductions is validated by simulations. The classification performance of the proposed technique is slightly reduced in terms of the packet error rate compared to perfectly known modulation schemes.

A framework of MAP algorithms for MC in OFDM-based communication systems with adaptive modulation is studied in [52]. This work extends the achievements in MAP-based MC [50,51] by adding a new constraint to the framework. In this paper, a metric approximation is used, whose accuracy increases with rising SNR; the reason behind using this is the high computational complexity of the optimal algorithm. The side information like the known frame structure, channel reciprocity, and the knowledge of total data transmission rate, which are typically available in wireless TDD systems are intensively utilized by the proposed classifiers. By utilizing this information, the proposed likelihood-based MC algorithms are highly effective for the short OFDM frames.

Another MAP-based MC for OFDM systems with adaptive coding and modulation (ACM) is carried out in [53]. The proposed classifier for QAM schemes utilizes the channel reciprocity in TDD systems that requires knowledge about the joint probabilities of the subcarrier-wise bit efficiencies at the transmitter and receiver sides. In contrast to prior heuristic approaches [52], these probabilities are calculated analytically if the transmitter and receiver apply the same bit loading (BL) algorithm on their erroneously estimated channel state information. Furthermore, the performance of the proposed MC algorithm employing analytical results is comparable to the simulated joint probabilities. However, it is still somewhat superior due to the subsidiary-independent technique’s sub-optimal approach [50]. Analytical and simulation results outperform the heuristic approach [52], especially at higher SNRs.

Another modulation classification algorithm for wireless TDD-based OFDM systems with adaptive modulation and coding is analyzed in [54]. The proposed MAP-based classifiers use the distinct signaling bits that are transmitted along with the information symbol. Thus, these can be viewed as a hybrid of MC and a signaling-based transmission principle. According to the signal structure of the received data symbols, these classification algorithms are characterized as bit allocation tables, i.e., a list of modulation formats used on each subcarrier. These received bit allocation tables are explicitly transmitted auxiliary information. Numerical studies indicate that the reliability of the classifier can be significantly enhanced by the use of the specified auxiliary information in a standard indoor propagation environment. Moreover, the simulation results of effective spectral performance show that the proposed method can be a reliable alternative in pure signaling-based or MC schemes in adaptive OFDM transmission. It outperforms the non-adaptive OFDM transmission system. However, this algorithm works in the presence of known CSI, knowledge about the total number of loaded bits, and coding rate.

An adaptive transmission algorithm for TDD-based wireless OFDM systems is carried out in [55]. In this technique, at the transmitter side, the BL algorithm and at the receiver side modulation classification algorithm are jointly optimized. To increase the effective data rate, a MAP modulation classification algorithm is applied in place of signaling the complete BAT to the receiver. The classification reliability is increased while preserving the enhanced link quality and low signaling overhead with this optimization on the BL algorithm. The idea behind this contribution is to maximize the effective bandwidth efficiency by this joint optimization of the BL algorithm at the receiver side and the modulation classification algorithm at the receiver side. Thus, the data rate loss caused by the signaling overhead is reduced. The simulations are performed in a typical indoor propagation scenario using burst transmission. It shows the enhancement of bandwidth and reduces the signaling overhead compared to the conventional methods.

A reciprocity-based MC algorithm for adaptive OFDM transmission systems in TDD mode is studied in [56]. This proposed transmission technique used the BL algorithm at the transmitter and MC at the receiver. A MAP-based MC is proposed, which is already effective for short frames if channel reciprocity in TDD systems is assumed. In this contribution, the authors analyze the performance of an improved version of this algorithm in a more realistic scenario. Simulations are carried out to validate the accuracy of the MC algorithm in the presence of imperfections caused by channel time-variance, channel estimation errors, and non-reciprocal transceiver filters. Simulation setup investigations are focused on indoor propagation scenarios typical for WLAN. For calibrated transceivers, the simulations show superior performance of the proposed adaptive transmission scheme with MC compared to a non-adaptive transmission in a typical indoor propagation scenario. It also has superior classification performance as compared to the signaling-based technique.

A simplified MAP-based MC is analyzed in [57]. An adaptive OFDM based on an IEEE 802.11a system is simulated. The system occupies a bandwidth of 20 MHz, which is split into N=64 subcarriers. Among these subchannels, $N_{d}=48$ subchannels are used for data transmission: 4 are reserved for channel tracking and synchronization purposes and the remaining 12 are unused. Throughout this paper, they have assumed perfect time and frequency synchronization, which they consider to be a typical indoor scenario. The number of multipath components is assumed to be 16 such that the length of the guard interval is set to be 16 too. The maximum Doppler frequency is assumed to be $f_{d}$ = 55 Hz corresponding to a speed of 3.33 ms, and the Doppler spectrum follows Jakes’ model. The correlation is very strong in the considered system due to the quantized structure of the effective channel. The quantization is a result of adaptive power allocation. In the context of this paper, a MAP-based MC approach is investigated in wireless local area network (WLAN) based OFDM systems with adaptive modulation. The receiver has to estimate the channel, which is modeled by a slowly varying multipath Rayleigh fading channel and AWGN. The performance of the MC algorithm is measured in terms of the end-to-end packet error rate (PER). Package errors occur due to data detection errors and MC classification errors. The PER of the proposed MC algorithm is almost identical to the PER of an error-free MC algorithm. This exemplifies the potential of MC applications in real-time scenarios.

Another MAP-based MC for the TDD-based OFDM system is studied in [58]. This paper proposes a channel prediction approach for improving the efficiency of the MC used in the adaptive OFDM scheme. To achieve an acceptable prediction performance, effective noise reduction and interpolation techniques are used. The channel is supposed to be frequency-selective, with Rayleigh fading coefficients and a power delay profile decided by the standard indoor environment for IEEE 802.11a models. For time correlation, Jakes’ Doppler spectrum is presumed, with the maximum Doppler frequency $f_{d}$ set to 20 Hz by default. Finally, simulations for the channel modeled with the Gaussian Doppler spectrum are carried out to explore the robustness of the proposed approach to the channel model. The probability of incorrect classification for both Jakes’ and Gaussian Doppler spectrum is compared, in the case $f_{d}$ = 20 Hz. In this case, it can be shown that the proposed technique is sufficiently robust to the model of the channel’s time variance.

The importance of adaptive modulation for effective usage of channel capacity in the OFDM system is shown in [59]. The need to transfer the information about modulation to the receiver is abolished by the MC algorithm, and thus, these algorithms are a very useful method to increase the channel capacity. However, in practice, two different sets of pilot symbols are used for the identification of the modulation type and for the estimation of the channel impulse response. The author proposes only one set of pilot symbols to find the information about the modulation type as well as the channel in this paper. As the pilot symbols are related to the modulation type, so they are named “adaptive pilots”. The identification of the modulation type is successfully done with the help of these adaptive pilots without affecting the performance of the channel estimation. By assigning unique pilots to every possible modulation type, the modulation information is embedded. BPSK, QPSK, 16-QAM, and 64-QAM are the possible pilot patterns with corresponding modulation types. It is shown by the simulation results that modulation types are successfully identified by the proposed adaptive pilots, while no effect is introduced to the channel estimation process. For the application of the proposed algorithm, pilots can be located at different locations with different values. However, when more number modulation formats are involved in the communication, more adaptive pilots may be required, which degrades the spectrum efficiency of the transmission.

The MC approach enables the estimation BAT technique in adaptive OFDM systems [60]. The authors analyze a less computationally complex MAP-based MC algorithm. They derive an estimation of the probability of classification error of a MAP-based classifier. Moreover, based on the derived estimation, a rate-adaptive (RA) BL algorithm is developed. The findings of the simulation reveal that the proposed RA algorithm greatly improves the accuracy of modulation classification. Furthermore, it is also shown that, in comparison to traditional RA methods, the proposed BL approach improves classification performance for SNR above 15 dB.

### 3.3. FB Approach

In the FB algorithm, the expert domain feature needs to be extracted first and then decisions are made for the classification. Some of the expert domain features are the variance of the normalized signal amplitude, phase, and frequency [101], the variance of the zero-crossing interval [102], moments, cumulants [63], cyclic cumulants [5], cyclostationarity [103], Fourier transform [63], wavelet transform (WT) [17], and constellation shape [104] of the received signal. The fuzzy logic [105], entropy [106], and constellation shape recovery technique also have been used for MC. Various decision-making approaches have been employed, including maximum-likelihood detector [63], Hellinger distance [107], Euclidean distance [108], and unsupervised clustering techniques [109].

#### MC with Higher Order Statistics (HOS)

Here, we provide a framework of the MC method with HOS [110]. The moment with the *k*th order and *p*th conjugations for $x_{m}$ associated with $x_{m}\left[n\right]$ is defined as

(13) $$M_{kp,x_{m}}=E\left[x_{m}^{k-p}{\left(x_{m}^{*}\right)}^{p}\right].$$

where ${\left(\right)}^{*}$ represents a complex conjugate. The corresponding cumulant with *k*th order and *p*th conjugations is defined as

(14) $$C_{kp,x_{m}}=cum\left(\underset{k-p}{\underset{︸}{x_{m},x_{m},\ldots ,x_{m},}}\underset{p}{\underset{︸}{x_{m}^{*},x_{m}^{*},\ldots ,x_{m}^{*}}}\right),$$

where cum() represents the joint cumulant function. HOS provides an integrated technique as well as a nonlinear signal processing viewpoint. Nevertheless, the information in the power spectrum of the second-order statistics is only appropriate for describing Gaussian processes statistically. In MC applications [111], a general fourth-order statistics $C_{42,x_{m}}$ is frequently used. According to (13) and the fourth-order cumulant formula for four random variables, *X*, *Y*, *Z*, and *W* can be expressed as

(15) $$cum\left(X,Y,Z,W\right)=E\left[XYZW\right]-E\left[XY\right]E\left[ZW\right]-E\left[XZ\right]E\left[YW\right]-E\left[XW\right]E\left[YZ\right],$$

and

(16) $$\begin{array}{cc} C_{42,x_{m}} & =cum(x_{m},x_{m},x_{m}^{*},x_{m}^{*})\\ & =E\left({\left|x_{m}\right|}^{4}\right)-{\left(\left|E(x_{m}^{2})\right|\right)}^{2}-2E^{2}\left({\left|x_{m}\right|}^{2}\right), \end{array}$$

In a similar fashion, a typical second-order cumulant can be written as

(17) $$C_{21,x_{m}}=E\left({\left|x_{m}\right|}^{2}\right).$$

The normalized fourth-order cumulant [12] is typically used to calculate MC, defined as

(18) $${\hat{C}}_{42,x_{m}}=\frac{C_{42,x_{m}}}{C_{21,x_{m}}^{2}}.$$

The FB-MC approaches are listed in Table 4, which highlights selected features, modulation types, channels, and undefined parameters.

Multicarrier modulation given by the OFDM signal generator using an IEEE 802.16e standard is studied in [61]. Based on the standard of IEEE 802.16e, three possible modulation formats can be used, such as QPSK, 16-QAM, and 64-QAM. The mean, variance, skewness, kurtosis index, and moment order of the received signal are all considered and compared in order to determine the modulation scheme non-line-of-sight (NLOS) with six multipath components. The dominant statistic features capable of separating the QPSK modulation scheme from 16-QAM and 64-QAM are skewness, kurtosis, and variance, as determined by the statistical properties of the received signal. Furthermore, the high order moment is one of the most important statistical features that distinguish the 16-QAM modulation scheme from the 64-QAM modulation scheme. However, in the context of timing and frequency synchronization issues, this approach does not perform well.

In another FB-MC [62], the amplitude moments and correlation properties are used to classify the modulation scheme for OFDM systems. This technique considers the presence of CFO, which is the cause of intercarrier interference (ICI) in the amplitude moments of the received signal. Therefore, the ICI component is estimated by using the correlation between the subcarriers. To determine the influence of ICI components in the amplitude moments, the authors derive the amplitude moment in the form of infinite series of elementary functions. It is observed that the amplitude moments increase as the frequency offset increases. Considering 4096 subcarriers in an OFDM symbol, at least 10 OFDM symbols are required to achieve the desired classification accuracy at 30 dB SNR. This approach outperforms the existing amplitude moment-based approach with the prior information about CFO. This is due to the estimation and elimination of the ICI components in the amplitude moments. However, this MC algorithm is restricted to known CSI and proper synchronization circumstances.

**Figure 3:**
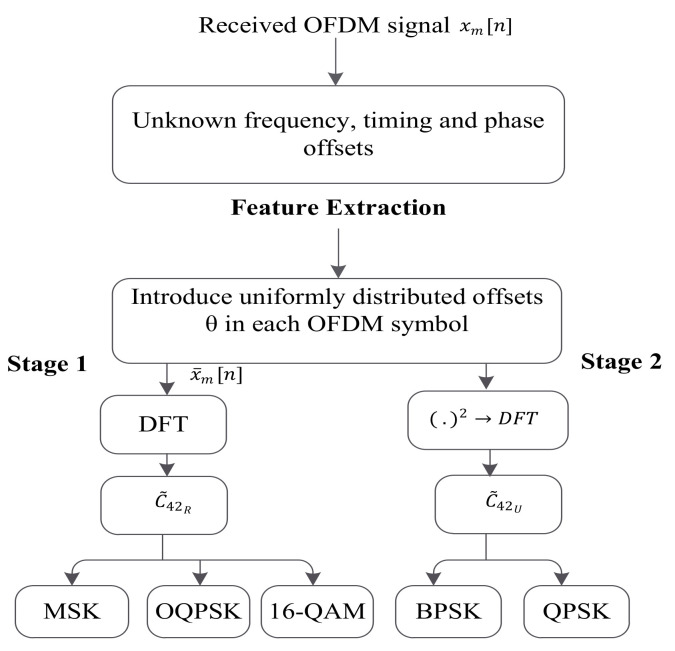
Schematic diagram of blind modulation classification studied in [63].

In [63], another FB blind MC approach is suggested and implemented on radio frequency (RF) testbed for OFDM signals. The authors use the combined features of DFT and the fourth-order cumulant, as shown in Figure 3. This algorithm does not need prior information about the signal parameters and CSI. It also works effectively when there are synchronization problems, such as timing, frequency, and phase errors. Before the feature extraction process, a random uniformly distributed timing offset is added in each OFDM symbol to reduce the influence of the timing offsets. The authors have listed BPSK, QPSK, OQPSK, MSK, and 16-QAM for the OFDM signal. The number of subcarrier N=1024, CP length $N_{cp}=N/4$, channel tap L=4, number of OFDM symbol 50, normalized CFO −0.5<ϵ<0.5, symbol timing offset [−N/2,N/2], sampling rate 50 Msamples/s, symbol rate 1 Msymbols/s, and Rayleigh channel are considered the simulation parameters for this technique. Classification is carried out in two stages. First, the received signal is transformed into the frequency domain by using the DFT operation, then the normalized fourth-order cumulant of the frequency domain signal is calculated. The modulation formats OQPSK, MSK, and 16-QAM can be distinguished by the normalized fourth-order cumulant, which is expressed as

(19) $${\tilde{C}}_{42_{R}}\;=\;\frac{1}{K}\;\sum_{m=1}^{K}\;\frac{{\vec{C}}_{42_{X_{m}}}\;-\;{\left|\frac{1}{K}\sum_{v=0}^{K-1}e^{-j4\pi v/K(\tau +\theta_{u})}X_{m}^{2}\left[v\right]\right|}^{2}}{\frac{1}{K}\sum_{v=0}^{K-1}{\left|X_{m}\left[v\right]\right|}^{2}-C_{21,W}},$$

where $X_{m}\left[v\right]$ represents the DFT of the received signal $x_{m}\left[n\right]$, $C_{21,W}=\sigma_{W}^{2}$ represents the estimated variance of AWGN, and *K* is the total number of OFDM symbols.

**Figure 4:**
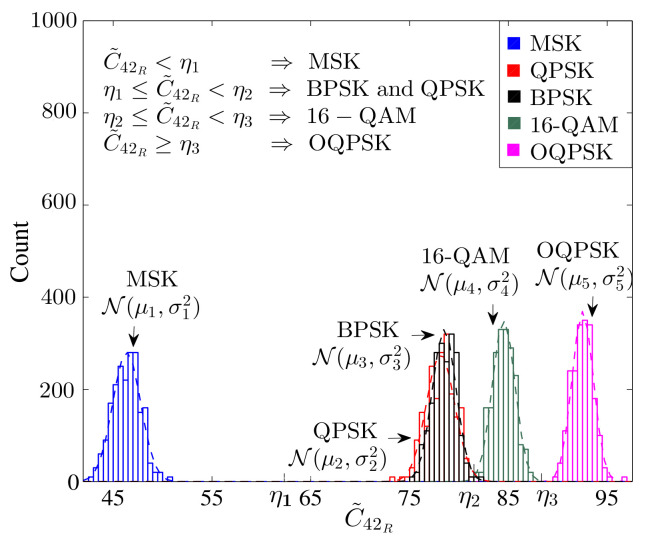
Histogram of ${\tilde{C}}_{42_{R}}$ for BPSK, QPSK, OQPSK, MSK, and 16-QAM. Adapted with permission from Ref. [63]. Copyright 2021 IEEE.

**Figure 5:**
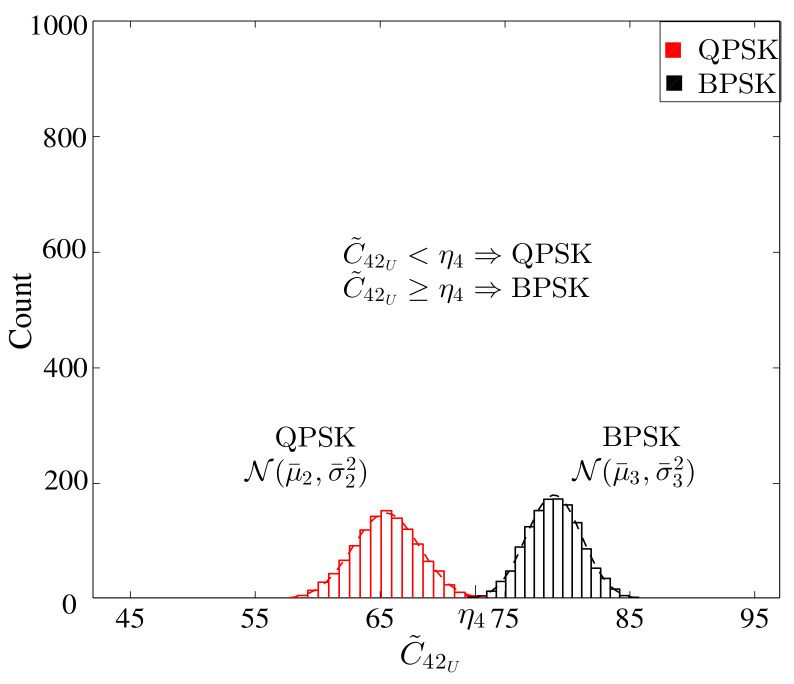
Histogram of ${\tilde{C}}_{42_{U}}$ for BPSK and QPSK. Adapted with permission from Ref. [63]. Copyright 2021 IEEE.

The histogram of the above is given in Figure 4. The second stage performs the DFT of the square of the received signal then calculates the normalized fourth-order cumulant, which is expressed as

(20) $${\tilde{C}}_{42_{U}}\;=\;\frac{1}{K}\;\sum_{m=1}^{K}\;\frac{{\vec{C}}_{42_{U_{m}}}\;-\;{\left|\frac{1}{K}\sum_{v=0}^{K-1}e^{-j4\pi v/K(\tau +\theta_{u})}U_{m}^{2}\left[v\right]\right|}^{2}}{\frac{1}{K}\sum_{v=0}^{K-1}{\left|U_{m}\left[v\right]\right|}^{2}-C_{21,W}},$$

where $U_{m}\left[v\right]=X_{m}\left[v\right]⊛X_{m}\left[v\right],⊛$ denotes the linear convolution operator. For BPSK and QPSK modulation schemes, the above Equation (20) gives different values, as shown in Figure 5.

In the paper [64], the authors use a method for applying wavelet transform (WT) to OFDM and SC signals to extract their transient characteristics and then use the transient characteristics to identify the two types of signals. Good performance can be achieved in low SNR and multipath channel conditions. In addition, the effects of the sample rate and symbol rate on the identification algorithms are analyzed and simulated. The author conducts a variety of simulation experiments to assess the performance of the proposed identification algorithms and the effects of the sample rate and symbol rate on the identification algorithms. All results are based on 100 Monte Carlo trials. The percentage of correct classification (PCC) versus the SNR plot represents the average variance of signals versus SNR. Every 8000 data samples make up of a trial source. We notice that the average variance is large in the OFDM curve and small in the SC curve. The result is well separated between OFDM and SC modulations. When SNR is 0 dB, the PCC between OFDM and SC signals can reach 100% when the symbol rate is greater than 20 kHz.

Another FB-MC [65] is using spectrum analysis to classify the OFDM and SC. The authors utilize the energy distribution parameter and the kurtosis of the power spectrum coefficient to classify OFDM and SC. This method does not need any prior information about the symbol rate, carrier frequency, etc. In simulation results, it is found that extracted spectrum parameters have better performance over AWGN as well as Rayleigh fading channels. It has a classification rate of up to 97% with an SNR at 10 dB.

In [66], the peak-density clustering algorithm is used to investigate an MC technique for adaptive optical OFDM systems. The clustering technique is used to find the centers of the signal constellation clusters. The number of cluster centers is calculated using the density and distance metrics of samples. The number of cluster centers is utilized to distinguish M-QAM. The OFDM signals are fed into an arbitrary waveform generator (AWG) with a sampling rate of 50 GSamples/s. The electrical OFDM signal is then converted into an optical signal using an external cavity laser (ECL) and an intensity modulator. The modulated optical signal is then routed through a variable optical attenuator (VOA) and an erbium-doped fiber amplifier (EDFA) to alter the signal SNR and mimic different transmission circumstances. They use a 50 GSamples/s real-time oscilloscope to capture data and another VOA to regulate the input power before the photodetector. Finally, OFDM MC and demodulation are conducted for a test sample of 8192 lengths in each optical SNR.

In the paper [67], the identification of these orthogonal modulations, i.e., OFDM, code division multiple access (CDMA), is studied. The classification method is based on general orthogonal modulations, whose modulation parameters should be estimated. The identification method applies to both adaptive modulation and increased security in radio communications. General orthogonal modulations are employed to identify modulations. First, the modulation parameters of rotation planes and angles are estimated. Orthonormal vectors are derived by received signal samples and rotated to hold orthogonality among time slots. Then, the inverse rotation corresponds to the modulation parameters to be estimated. The difference vector between the received signal vectors is used for this method. In computer simulations, OFDM, CDMA, a block of QAM, and so on are considered candidate modulations. The bit error probability of the estimated modulation is presented to compare the performance from the point of view of SNR and the number of samples. The proposed estimation performance is evaluated in the AWGN channel by computer simulations.

Based on the higher-order cumulants, an MC algorithm is carried out that discriminates the OFDM signals from SC signals [68]. First, OFDM signals are discriminated from SC signals based on distinct features parameters over the Rayleigh channel. In order to verify the effectiveness, the modulation set is assumed as OFDM, 2-FSK, 4-FSK, 8-FSK, BPSK, QPSK, 8-PSK, 16-QAM, 32-QAM, and 64-QAM. The combination of the second- and fourth-order cumulants is used as the feature to discriminate the OFDM signals from the SC signals. Simulation results show that the algorithm is stable with low computational complexity and high PCC in low SNR level.

In the paper [69], a classification technique is devised for identifying the OFDM signals from the SC. In addition to differentiating the OFDM signals from SC, some important parameters of OFDM signals are estimated for further processing. The estimated parameters include the number of subcarriers, the length of the OFDM symbol, and the CP length. Using these parameters, traditional modulation classification techniques may be used to identify the linear modulation format on each OFDM subcarrier. The analytical distribution function-based Gaussian test technique is shown to differentiate OFDM from SC modulations effectively, and the correlation test is shown to estimate the cyclic prefix length effectively. A fast Fourier transform (FFT) is used to effectively estimate the number of subcarriers. The simulation findings show that the proposed technique provides classification performance of more than 90% for SNR greater the 15 dB.

In the paper [70], a Bayesian inference-based MC technique for the MIMO-OFDM signal is used. This technique uses the Gibbs sampling convergence approach on a latent Dirichlet model as a baseline. However, the inference-based technique has a significant computational overhead, and it also needs perfect synchronization at the receiver.

In the paper [71], an MC algorithm for the MIMO-OFDM system is analyzed under the unknown frequency-selective fading channels and SNR. This work is an extension of the achievements in MAP-based MC [50,51] by adding a new constraint to the framework. The classification problem is presented as a Bayesian inference task, with solutions provided based on Gibbs sampling and mean-field variational inference. The Gibbs sampling method yields the best Bayesian result. It is shown that after multiple iterations, switching to the mean-field variational inference technique improves classification accuracy for the small length of the received signal. However, most of the existing MC consider channels as flat fading when the number of receiving antennas exceeds the number of transmitting antennas. However, under more general circumstances, the proposed algorithm works quite well. It is shown that the proposed Bayesian methods outperform existing non-Bayesian techniques based on independent component analysis (ICA). However, this inference-based technique is quite difficult, and it also necessitates perfect synchronization at the receiver.

A tree-based blind MC method for asynchronous MIMO-OFDM is developed in [72]. It extracts unique features for different modulation schemes using normalized fourth-order and sixth-order cumulants. It then performs a threshold-based classification using the likelihood ratio test to determine the modulation format of the received signal. The number of subcarrier N=128, CP length $N_{cp}=N/4$, number of channel tap L=4, number of OFDM symbol 50, normalized CFO −0.5<ϵ<0.5, symbol timing offset [−N/2,N/2], sampling sampling rate 50 Msamples/s, symbol rate 1 Msymbols/s, and Rayleigh channel are considered the simulation parameters for this technique. The classification performance of this algorithm is validated by using the RF testbed in a realistic scenario. The authors consider the higher number of transmitting and receiving antennas in the simulation process. However, the actual experimental systems in this paper only contain at most two transmitter antennas and two receiver antennas.

In the paper [112], signal parameter estimation, modulation classification, and synchronization are carried out for the OFDM signal. At the first stage, the cyclic cumulant is used to estimate the number of subcarriers, symbol length, useful symbol length, CP length, and oversampling factor. At the second stage, the elementary cumulant is used to classify the BPSK, QPSK, OQPSK, MSK, and 16-QAM modulation scheme over the Rayleigh fading channel. After that, a modified maximum likelihood technique is used to estimate the CFO and STO for the OFDM system jointly. After correction of the CFO and STO, recovery of the constellation diagram of modulation schemes and BER analysis is performed. The BER is found approximately $8.5\times 10^{-3}$ and $6.5\times 10^{-2}$ at 20 dB SNR for QPSK and 16-QAM modulation schemes, respectively. This technique is also validated over the NI RF testbed setup over an indoor propagation environment.

## 4. Artificial Intelligence-Based Approach to MC

AI is certainly the next big “game-changing” technology that includes both ML and DL. In MC, ML finds lots of significance in terms of decision trees, KNN, support vector machine (SVM), artificial neural network (ANN), and some hybrid algorithms. DL is a kind of a subsidiary of ML, which originates from the study of ANN. Neural networks are inspired by biology and try to mimic the neural structure of the human brain [113,114]. Recently, researchers in the field of wireless communication stated using DL extensively. It finds applications especially in the field of communication systems, such as non-orthogonal multiple access (NOMA) technology, MIMO technology, resource allocation scheme, and signal MC. Table 5 and Table 6 lists a few of the ML- and DL-based MC algorithms studied in the literature, outlining the modulation types, uncertain parameters, and the channel employed.

### 4.1. ML-Based MC

In this paper [73], the authors extract the features by calculating higher-order cumulants, then the extracted features are applied to naïve Bayes classifier for MC. However, the authors assume proper equalized and perfectly synchronized signals received at the receiver. The features, combinations of fourth-order $C_{42}$ and sixth-order cumulants $C_{63}$ often produce better classification performance than using each of these features alone. By using the same set of features, the naïve Bayes classifier is compared with the ML-based classifier and SVM-based classifier. It is observed that the naïve Bayes classifier outperforms the ML-based classifier and SVM-based classifier with less computational complexity.

This paper [74] introduces a technique for classifying OFDM signals using higher-order moments and cumulants with multiple types of classifiers and cluster techniques. There are four considered methods of classification, namely, KNN, ML, SVM, and neural network (NN) classifiers. Fuzzy *k*-Means and fuzzy *c*-means are two cluster techniques that are used for the two classes of OFDM signals. One class is considered fixed WiMAX (IEEE 802.16d), which includes BPSK, QPSK, 16-QAM, and 64-QAM modulations. Another class is considered OFDM signals used in Wi-Fi (IEEE 802.11a), which includes and BPSK, QPSK, 16-QAM, and 64-QAM modulations. In the simulations, the input signals are normalized to have zero mean and unit variance after transmitting through the Rayleigh fading channel. The normalized output signal is then used for the feature extraction process. Higher-order moments and cumulants up to the 8th order are used to extract features. The extracted features are used as input to the different types of classifiers, such as SVM, KNN, ML, and NN classifiers, which use the fuzzy *k*-means and fuzzy *c*-means as clustering techniques. The performance of the SVM classifier with the fuzzy *k*-mean is better than all the combinations of classifiers and clustering algorithms for most of the SNR values.

The MC problem for MIMO systems employing OFDM under imperfect timing synchronization scenarios is studied in [75]. The proposed algorithm first uses the HOC of the received signal to extract the unique features, which show the robustness to STO. After that, a random forest classifier is used as the decision criterion to perform the classification problem. The main benefits of random forests are their better classification performance and low exposure to noise. The number of subcarrier N=128, CP length $N_{cp}=N/4$, channel tap L=5, number of transmitting antennas 2, number of receiving antennas 8 and frequency-selective channel are considered the simulation parameters. The simulation results show that the proposed classifier can work well in the presence of STO with satisfactory classification accuracy. In a realistic scenario, where perfect STO estimation is difficult to achieve, these algorithms can provide conceptual help.

In the paper [76], a modulation classifier without knowing noise variance is studied for the OFDM system. In order to estimate the amount of phase rotation caused by flat fading, the authors investigate adopting the iterative closest point, which is a kind of template matching technique. Combining the least squares-based phase estimation, the classification performance of the proposed method can be improved significantly. The PCC at several SNRs when each correction is performed in flat fading, where four types of modulation schemes, i.e., BPSK, QPSK, 16-QAM, and 64-QAM, are used. From these results, it is found that, as compared with the method of using only the least-squares method, the method combining the least squares (LS) method with the iterative closest point (ICP) algorithm does not deteriorate the accuracy of the phase correction, even if the number of signal points decreases.

In this paper [77], the classification of OFDM, BPSK, QPSK, Gaussian frequency-shift keying (GFSK), 16-QAM, and 64-QAM is realized by MATLAB programming based on the characteristic of HOCs. A new feature parameter is proposed according to the second- and sixth-order cumulant. Simulations are conducted with classifiers, including KNN, SVM, decision theory, and back-propagation neural network (BPNN). It is found that the average classification rate is greater than 95%.

In the paper [78], the authors propose a blind MC algorithm for space-time block coding (STBC)-based MIMO-OFDM system, which works in the presence of CFO, channel estimation errors, and impulsive noise. Multiple signal classification (MUSIC) algorithms are used to estimate the CFO and channel statistics. The estimated CFO and channels are compensated and equalized, then features are extracted using higher-order moments (HOMs) and HOCs. Finally, the extracted features are applied to ANN, SVM, RF classifier (RFC), and KNN classifier. The simulation results show that the SVM and ANN classifiers have better classification performance, even at low SNR.

In [79], an SVM-based MC algorithm is studied for the OFDM system in the presence of frequency offset in which statistics-based features are used as input of the SVM classifier. The number of peaks in the distribution of amplitude, the variance of the amplitude, the variance of the phase, and the variance of the spectrum are extracted from the received signal. These extracted features are used to make a dataset. This dataset is applied to the SVM classifier to classify the modulation scheme of the received signal. The proposed method shows great accuracy in high SNR channels with over 80% accuracy. It also shows robustness against the frequency offset. However, when the signal is flooded by noise and extremely influenced by frequency offset, the proposed still has over 50% accuracy. The algorithm is tested experimentally on the SDR platform, which can realize a variety of communication systems by updating the software. Based on such a popular SDR hardware platform and using GNU Radio, the modulation formats are generated, transmitted, and classified.

In [80], the design and implementation of the MC algorithm for the OFDM visible light communication (OFDM-VLC) system are explored. Clustering and Gaussian model analysis are used to obtain the classification feature values. The modulation format is then classified using these classification feature values. The simulation results show that the suggested method can achieve 100% classification accuracy at 1 dB to 2 dB lower than that of the clustering scheme. Furthermore, the experimental findings show that the suggested MC technique is feasible in an OFDM-VLC system.

In [81], an MC algorithm base on a hierarchical iterative SVM classifier is studied for the OFDM signal. To extract characteristic values from OFDM signals, higher-order cumulants and bi-spectral envelope peaks are used, and the resulting characteristic values are then processed to create fresh training sample data. The feature extracted by using HOCs is used to distinguish multicarrier signals from the SC signals. The bi-spectral envelope peaks are used to distinguish the OFDM signal from the multicarrier signal. The training dataset obtained from the higher-order cumulants and bi-spectral envelope peaks of the received signal is applied to the input of a hierarchical iterative SVM classifier. The number of subcarrier N=128, CP length $N_{cp}=N/4$, symbol rate 1024 bps, sampling rate 3000 kHz and Rayleigh channel are considered the simulation parameters for this technique. It is found the classification accuracy of the SVM-based classifier is improved when compared with the wavelet transform method and higher-order cumulant-based method.

### 4.2. DL-Based MC

A lot of focus has recently been drawn by DL due to its effective ability to integrate offline preparation and online deployment [115]. DL is a specialist in automated feature extraction from a huge amount of data instead of the complicated and challenging nature of man-made features [116,117].

In the paper [82], a cepstral algorithm for MC is proposed with adaptive modulation in OFDM systems. The expert domain features of the received signal are extracted using Mel frequency cepstral coefficients (MFCCs), and the modulation formats and their order are classified using a multi-layer feed-forward neural network. This classifier has the capability of recognizing the M-ary amplitude-shift keying (M-ASK), MSK, M-PSK, M-ary frequency-shift-keying (M-FSK), M-QAM signals and the order of the identified modulation. The classification performance of the proposed technique is evaluated using the false classification probability (FCP). The AWGN channel is taken into account when creating the mathematical model for most of the results. The simulation results reveal that the modulation format and order can identify by extracting cepstral features from the received signal and with the help of the transforms, such as discrete cosine transform (DCT), discrete sine transform (DST), and the discrete wavelet transform (DWT). These classify the distinct features using a robust back-propagation feed-forward neural network for different modulations, such as QPSK, 8-QAM, 16-QAM, 32-QAM, 64-QAM, and 128-QAM. The proposition to identify the modulation type and order is proven to be considered effective.

In the paper [83], the authors develop an MC algorithm that is based on the bispectrum and CNN AlexNet models. As we know, bispectrum is a high-order statistic that suppresses AWGN well and is frequently utilized in signal detection and nonlinear system characterization areas. Furthermore, AlexNet exhibits outstanding image classification performance despite having a very basic structure of eight layers. First, the authors compute the bispectrum of received signals, then take the amplitude spectrum of the bispectrum (ASB), which is used as input to the CNN network. After that, they fine-tune the chosen AlexNet, which automatically extracts the distinct features from ASB images. Finally, these features are passed into a softmax classifier, which classifies the modulation type. The simulations are performed under different noise environments for the dataset that includes BPSK, 2-ASK, 2-FSK, 4-FSK, 8-FSK, linear frequency modulation (LFM), and OFDM signals. It is observed that the bispectrum-AlexNet model has a classification accuracy greater than 97.7% when the SNR is greater than 5 dB.

The above MC techniques are developed by utilizing feature extraction-based machine learning. Moreover, the standard approaches face bottlenecks, where the PCC is very small and it is also impossible to incorporate them in realistic OFDM systems because it is difficult to extract distinct features from OFDM signals using conventional methods. In order to address this problem, the authors [84] suggest a CNN-based MC system for recognizing OFDM signals. In particular, a CNN is used to train in-phase (I) and quadrature (Q) samples for OFDM signals. The authors construct two datasets with separate modulations for the MC function. Dataset 1 contains BPSK, QPSK, 8-PSK, and 16-QAM modulations, while dataset 2 contains BPSK, QPSK, 8-PSK, 16-QAM, and 64-QAM modulations. These two datasets are utilized to test the robustness of the proposed system. Each modulation format considers 20,000 data samples for training and research. Since CNN can efficiently extract the distinct features of received OFDM signals, the simulation results indicate that CNN trained on I and Q samples achieves better classification performance than conventional machine learning based approaches.

**Figure 6:**
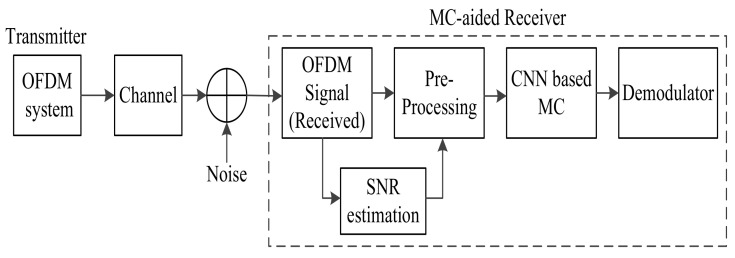
Framework of the proposed CNN-based MC system [85].

**Figure 7:**
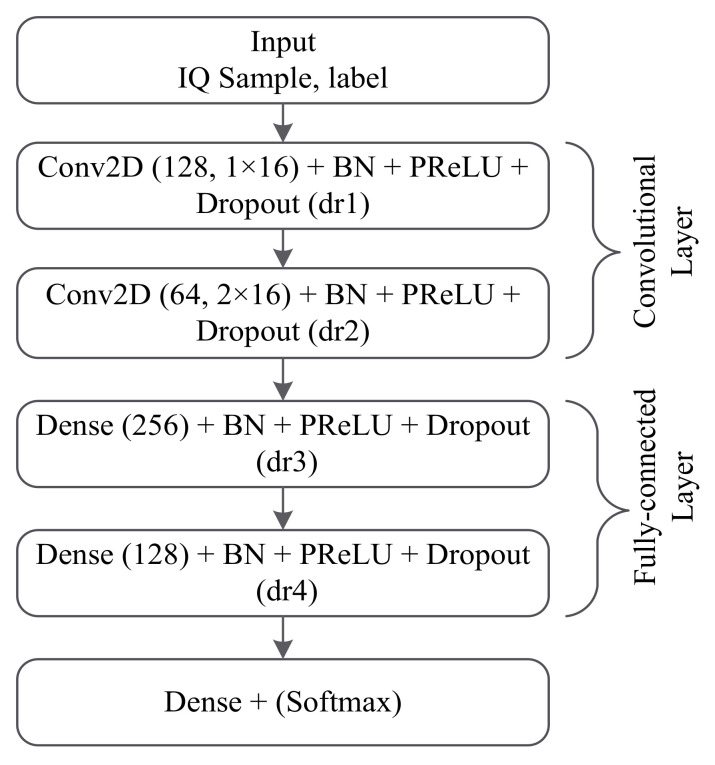
CNN structure design in the proposed CNN-based MC method [85].

In the paper [85], a CNN-based MC method by considering the phase offset effect is studied. As shown in Figure 6, CNN-based MC is first trained by the received I and Q samples in the presence of phase offsets at different values of SNR. As shown in Figure 7, the DL-based MC technique is mainly implemented by CNN. The PReLU is used as an activation function for all layers, except the last layer, and the softmax is used as an activation for the last layer to implement a multi-classification problem. The authors use two sets of data with different modulation modes for the MC issue, i.e., Dataset 1 and Dataset 2, to verify the robustness of the classification technique [85]. The number of subcarrier N=16, CP length $N_{cp}=2$, number of OFDM symbol 6 and AWGN channel are considered the simulation parameters for this technique. Comparative experiments show that its performance of classification is much higher than the conventional extraction methods. Moreover, the classification accuracy relatively reduces at the low SNRs due to the presence of phase offsets. By gradually increasing the SNRs, effective classification accuracy can be achieved eventually.

An adaptive modulation model based on machine learning for a MIMO-OFDM system is carried out in [44]. The 5G new radio (NR) technology can be used in a wider range of internet of things (IoT) applications than traditional systems. The adaptive modulation technique, which changes data rate and latency based on channel conditions, can be efficiently employed in 5G digital NR technology. The traditional adaptive modulation technique is developed by assigning modulation formats based on the channel conditions since the rule-based MC is unable to analyze transmission efficiency based on channel correlations between antennas. The number of propagation modes is enhanced exponentially based on the available number of modulations and antennas. So, these are not appropriate for 5G NR systems. The proposed adaptive modulation technique is learned by the training data, which are generated by the feature extracted from the received signal. The DNN application for adaptive modulation is the primary method of the main component analysis, which improves the model efficiency. The simulation results on the optimal transmission mode classification for the MIMO-OFDM signal show that the proposed model supports adaptability according to the condition of the complex MIMO channel.

In [86], the authors here present a CNN-based MC approach for the identification of OFDM signals, which is linked to a CNN that is trained on I and Q samples. The suggested CNN-MC technique is made up of two parts: three convolutional layers and four fully connected layers. The number of subcarrier N=16, CP length $N_{cp}=2$, number of OFDM symbol 6 and Rician channel are considered the simulation parameters for this technique. The suggested technique outperforms existing modulation classification algorithms in terms of accuracy and reliability. However, any parameters, such as number of subcarriers, number of null subcarriers, STO, CFO, phase offset, and CP length, change these MC and do not provide accuracy of more than 50% for adaptive OFDM systems.

The other approach described in [87] focuses on two-step training to enhance the classification performance of CNN-based classifiers. Transfer learning is also introduced to increase the performance of the retraining. A wider range of modulation formats for the OFDM signal, such as BPSK, QPSK 8-PSK, 16-QAM, 32-QAM, and 64-QAM, is recognized by the suggested technique.

In [88], the MC algorithm for the OFDM signal is developed by using an intelligent pyramid model. This algorithm has four stages, i.e., pre-processing, feature extraction, feature clustering, and classification. In the pre-processing step, the authors improve the received signal quality, which involves two steps quality evaluation and quality augmentation, using the bi-fold signal fortification (BFSF) approach. The number of subcarrier N=2048, CP length $N_{cp}=3$, sampling frequency 5 MHz and AWGN channel are considered the simulation parameters for this technique. If the received signal quality is poor, then quality augmentation is performed, taking into account noise reduction, equalization, quantization, and CFO compensation. Then the feature extraction process is performed by the gated feature pyramid network (GFP-Net). After that, the authors make the cluster from the extracted feature by using an intelligent twin-functioned human mental search (TF-HMS) optimizer to minimize the classification complexity. Finally, they offer the multi-distance-based nearest centroid classifier (MDNCC) technique, as well as improved Q-learning (IQL), to determine the correct modulation format for the received signal. However, this technique only considers the CFO as the synchronization when performing the modulation classification of the received signal.

In [89], a CNN long short-term memory (CNN-LSTM) based dual-stream structure for MC is developed. The first stream extracts local raw temporal characteristics from raw signals, while the second stream learns knowledge from amplitude and phase data. To learn spatial and temporal information from each stream, CNN-LSTM is used, which combines the spatial feature extraction ability of CNN and superior capacity of processing time-series data of LSTM. Furthermore, the features learned from two streams interact in pairs as a result of an effective operation, expanding the diversity of characteristics and, therefore, improving the classification performance of the received signal.

In [90], a CNN-based MC is studied in order to classify SC and OFDM systems with varying symbol lengths. The majority of older DL-based MC algorithms misinterpreted OFDM-based signals with varying OFDM usable symbol lengths. To address this issue, FFT window banks (FWB) are utilized as input to the CNN model to estimate the length of an OFDM symbol. After estimating the OFDM symbol length, a CNN-based MC technique is utilized to categorize the OFDM and SC modulation formats concurrently using FWB and IQ samples as combined input. However, compared to the traditional DL-based MC, this technique needed a longer received symbol to obtain the correct classification.

In [91], an OFDM signal identification technique based on a hybrid grey wolf optimization (HGWO) algorithm to optimize with a deep neural network model is carried out. This technique can distinguish the OFDM modulation signal from complex signals, such as SC, OFDM signals, and wavelet packet signals (WPM) in a multipath channel. In this technique, mixed order moment $u_{20}=M_{42}\left(x_{m}\right)/M_{20}^{2}\left(x_{m}\right)$, characteristics parameter $R=\sigma^{2}/\mu^{2}$, and $N=\frac{BW}{\Delta f}-1$ are extracted from the received signal. Then, a dataset is prepared by using $u_{20}$, *R*, and *N*, which are the input of the classifier. Then the HGWO algorithm is used to optimize the weights and thresholds of the DNN. The experimental findings demonstrate that the suggested method significantly speeds and improves the convergence speed of GWO. When compared to traditional methods, such as particle swarm optimization (PSO) and whale optimization algorithm (WOA), this technique outperforms the two. However, the HGWO technique in this study is limited because it is only used to improve the weight and threshold of the DNN model, and the network structure must be chosen manually. The intelligent optimization technique applied to the structure of the deep learning network may enhance classification accuracy.

In [92], the MC technique for OFDM VLC systems based on transfer learning (TL) is developed. For virtually all SNR values, the suggested AlexNet/GoogLeNet-TL-based strategy outperforms previous approaches in which the AlexNet/GoogLeNet is trained from scratch (AlexNet/GoogLeNet-SC). In more practical, few-training-data circumstances, AlexNet/GoogLeNet-TL outperforms AlexNet/GoogLeNet-SC by a wide margin.

In [93], the authors design and implement lightweight CNN (LCNN) based MC methods, i.e., the ShuffleMC method for the IoT cyber–physical systems. The ShuffleMC technique requires considerably fewer parameters and is far less computationally complex than the CNN-based MC method but the classification performance of both is almost the same at high SNR. Furthermore, the authors introduce the FFT to pre-process the received OFDM signals for improved classification performance and training acceleration. In addition, $l_{2}$ regularization is used in the training procedure to minimize over-fitting and marginally enhance classification performance.

In [94], a hierarchical CNN-based MC is developed for the waveform and MC in radar communications systems. Using time-frequency representation of the received signal from the Fourier synchrosqueezing transformation (FSST) and deep CNN, the received signal is categorized as either SC radar signals or multicarrier radar signals. Then the cyclic prefix duration, the number of subcarriers, and subcarriers spacing are estimated for the received OFDM signal. After that, the independent component analysis (ICA) operation is used to make the I- and Q-components, which are fed into the CNN classifier for MC.

In [95], a spectrum interference-based two-level data augmentation method in CNN for MC is studied. The short-time Fourier transform (STFT) and IFFT are used to assist in the expansion of signals and the introduction of variations while maintaining the key characteristics. The frequency-domain data are provided to radio signals to improve modulation classification. Experimental results demonstrate that using a two-level data augmentation approach based on spectrum interference may considerably enhance the accuracy of the deep CNN for MC, especially when the SNR is low. This methodology obtains state-of-the-art classification accuracy when compared to a range of data augmentation approaches and leading modulation classification algorithms using the public dataset RadioML 2016.10a.

In [97], a CNN-based MC algorithm is designed, which used a novel data generation technique allowing deep networks to compute correlations between samples inside each OFDM symbol and between symbols. The authors construct a unique advanced processing block that integrates attention and residual connections to boost the learning efficiency of the model. This approach is tested on a synthetic OFDM signal dataset and shows improved classification performance under various channel circumstances.

The cross-talk between sub-carrier has been addressed in terms of CFO. The errors in CFO destroys the orthogonality among the subcarriers or subchannels, thereby introducing ICI. Therefore, classification performance for the MC algorithm may degrade due to ICI or the presence of CFO. Therefore, we need to estimate and compensate for the CFO before MC [63]. In the paper [62], the amplitude moments and correlation properties are used to classify the modulation scheme for OFDM systems. This technique considered the presence of CFO, which is the cause of ICI in the amplitude moments of the received signal. Therefore, the ICI component is estimated and eliminated by using the correlation between the subcarriers. This approach achieves the desired classification accuracy at 30 dB SNR for the normalized CFO for range 0.1≤ϵ≤0.2. In [63], the authors use the DFT and fourth-order cumulant to classify the modulation scheme in the presence of CFO. However, this technique has good classification accuracy for the normalized carrier frequency offset of range −0.5≤ϵ≤0.5. In [90], a CNN-based MC is studied to classify modulation format for OFDM systems in the presence of CFO. FFT window banks (FWB) are utilized as input to the CNN model to estimate the length of an OFDM symbol. After estimating the OFDM symbol length, a CNN-based MC technique is utilized to categorize the OFDM and SC modulation formats concurrently, using FWB and IQ samples as combined input. The classification performance of this technique degrades to 87.3% in a minor CFO and 83.4% in a moderate CFO. However, it has a classification accuracy of 98.5% at high SNR in the absence of CFO.

## 5. Challenges and Future Research Directions

Based on an exhaustive literature review, this paper summarizes the two major MC approaches for the OFDM signal: statistics based and AI based, and also highlights their advantages and disadvantages. In the statistics-based approach, the LB approach provides optimal classification performance. As the number of unknown parameters increases, it becomes more computationally complex to find a desired analytical solution for the decision problem. If there is a closed-form solution made, it can be impractical because of its high computational complexity. A sub-optimal classifier is obtained from the optimal ML classifier to minimize computational complexity. In the FB algorithm, the expert domain feature needs to be extracted first, then decisions are made for the classification. FB algorithms are easier to implement, despite being sub-optimal. Many of the ML- and DL-based MC first use the signal pre-processing step, which includes noise reduction, parameter estimation, and making the signal synchronized, which enhances the quality of the received signal. After that, proper selection of classification model that can reduce the signal processing steps, increase the modulations classification accuracy and provide more reliable and effective methods of modulation classification, compared to conventional modulation methods.

Nevertheless, several studies are mainly based on ideal hypotheses and rely on a large number of labeled signals. Most of the MC research is still focused on the simulation stage. The communication environment is more sophisticated, and signal frame lengths are varied in the realistic implementation scenario. However, with the increasing complexities of the communication environment and the increasing need for numerous particular tasks, it is difficult to make sure that a huge training data set is generated effectively for particular tasks. The development of semi-supervised algorithm systems is needed to solve this problem. Effective semi-supervised algorithms may be able to fulfill the increasing need for diverse signal processing demands by collecting a large amount of data, only a small fraction of which is labeled data. Another potential task is to figure out how to develop hardware platforms, implant applications, and evaluate algorithms employing measured data.

Another challenge in the future is how to incorporate a DL-based transmission signal modulation identifier for OFDM signals on a field-programmable gate array (FPGA), which would necessitate further research into data quantization, model compression, and other related studies. Finally, DL techniques have a wide range of applications and growth potential as a powerful method for processing data and extracting features. In various fields, combining the DL model with other intelligent algorithms will yield more efficient results. Furthermore, traditional DL-based MC is challenging to implement in OFDM-based narrow-band (NB)-IoT devices, as it requires high computational complexity and more power as well as memory resources. However, implementing light-weight DL-based blind MC for NB-IoT devices that need less computational, space, and power requirements might be a difficult task for future adaptive transceiver systems. Another challenge in the future is modulation classification for OQPSK, π/4-QPSK, and MSK. Higher-order modulation classification for OFDM, MIMO-OFDM system, and adaptive OFDM systems over a randomized environment using a hybrid model need to be proposed in future wireless communication. In addition, we have to extend to a large number of modulation formats that work for all types of systems. MC can be implemented for massive MIMO systems, such as intelligent reflective surfaces, to reduce the distortion due to the non-line-of-sight (NLOS) component of the signal in future wireless communication.

In OFDM-IM, the number of active subcarriers can be adjusted to achieve the desired spectral efficiency and BER performance. Thus, the MC algorithm for OFDM-IM needs to be explored. As compared to the OFDM system, the filter bank multicarrier (FBMC) system does not require a CP, so it makes the use of radio resources more efficient. Therefore, MC for FBMC can be a future problem. In NOMA, if a different user uses a different modulation format, then MC for NOMA can be a challenging task. As compared to the OFDM system, orthogonal time frequency space (OTFS) has significantly high error performance over delay-Doppler channels with a wide range of Doppler frequencies. MC for OTFS can be a future research problem for designing advanced wireless communication systems. Due to the high peak-to-average power ratio (PAPR), it is difficult to use OFDM on the uplink. To overcome this problem, single-carrier frequency division multiple access (SC-FDMA) is used on the uplink. Therefore, the MC algorithm for SC-FDMA needs to be developed. MC for multicarrier code-division multiple access (MC-CDMA) can also be a critical research problem for future wireless communication.

## Figures and Tables

**Table 1:** List of abbreviations in alphabetical order.

Acronym	Explanation
AI	Artificial Intelligence
ALRT	Average Probability Ratio Test
AMAP	Approximated Maximum a Posteriori
ASB	Amplitude Spectrum of Bispectrum
AWGN	Additive White Gaussian Noise
BAT	Bit Allocation Table
BFSF	Bi-Fold Signal Fortification
BICM-ID	Bit-Interleaved Coded Modulation Iterative Decoding
CNN	Convolutional Neural Network
CSI	Channel State Information
DBN	Deep Belief network
DVB	Digital Video Broadcasting
FB	Feature Based
FCP	False Classification Probability
FFT	Fast Fourier Transform
FNSF	Frequency Non-Selective Fading Channel
FPGA	Field Programmable Gate Array
FSF	Frequency Selective Fading Channel
FSST	Fourier Synchrosqueezing Transformation
GLRT	Generalized Likelihood Ratio Test
HGWO	Hybrid Grey Wolf Optimization
HLRT	Hybrid Likelihood Ratio Test
HOC	Higher Order Cumulant
HOS	Higher Order Statistics
ICI	Inter-carrier Interference
IQ	In-phase and Quadrature
IQL	Improved Q-learning
KNN	K-Nearest Neighbors
KS	Kolmogorov–Smirnov
LLR	Log-likelihood ratio
MAP	Maximum a Posteriori
MC	Modulation Classification
MFCC	Mel Frequency Cepstral Coefficient
MDNCC	Multi-Distance-Based Nearest Centroid Classifier
NOMA	Non-Orthogonal Multiple Access
OFDM-IM	Orthogonal Frequency Division Multiplexing with Index Modulation
PCC	Percentage of Correct Classification
PDF	Probability Density Function
PER	Packet Error Ratio
PSO	Particle Swarm Optimization
SC	Single Carrier
SDR	Software-defined Radio
STFT	Short-Time Fourier Transform
TDD	Time Division Duplex
TF-HMS	Twin-Functioned Human Mental Search
UMP	Uniformly Most Powerful
VLC	Visible Light Communication
WOA	Whale Optimization Algorithm
WPS	Wavelet Packet Signals
WT	Wavelet Transform

**Table 2:** Summary of LB approaches for OFDM signals.

Author(s)	Classifier(s)	Modulation(s)	Parameter(s)	Channel	Average PCC at 20 dB SNR
T. Yucek [45]	Sub-optimum algorithm	BPSK, QPSK, 16-QAM and 64-QAM	Imperfect noise variance	AWGN	99.9%
J. Leinonen [46]	Quasi-log-likelihood Ratio Test based classifer	BPSK, QPSK, 16-QAM and 64-QAM	Known channel correlation between adjacent subchannels	AWGN	98.50%
J. Zheng [47]	ALRT, HLRT and Energy-based detector	BPSK, QPSK, 8-PSK and 16-QAM	Known CSI, Known noise variance and unknown CSI	Rayleigh	97.40%
T. Fang [48]	Expectation maximization block-quasi HLRT (EM-Block-QHLRT)	BPSK, QPSK, 8-PSK and 16-QAM	Unknown CSI and unknown noise power	Acoustic Rayleigh	100%
M. Marey [49]	Iterative EM-based MC algorithm, bit-interleaved coded modulation iterative decoding (BICM-ID) scheme	QPSK, 64-QAM, 1024-QAM and 8194-QAM	Presence of synchronization error and known and unknown CSI	Rayleigh	99%

**Table 3:** Summary of maximum a posteriori (MAP) based classifiers for OFDM signals.

Author(s)	Classifier(s)	Modulation(s)	Parameter(s)	Channel	Average PCC at 20 dB SNR
L. Häring [50]	MAP Algorithm, channel reciprocity in TDD systems	BPSK, 4-QAM, 16-QAM and 64-QAM	Perfect knowledge about data rate	Rayleigh	99%
L. Häring [51]	ML and MAP Algorithm	no modulation, BPSK, QPSK, 16-QAM and 64-QAM	Perfect synchronization and unknown CSI	Rayleigh	99%
L. Häring [52]	Simplified MAP algorithm that utilized frame structure, channel reciprocity, total number of transmitted data	no modulation, BPSK, QPSK,16-QAM and 64-QAM	Perfect knowledge about data rate	AWGN	100%
L. Häring [53]	Improved Approximated MAP Algorithm	QPSK, 16-QAM and 64-QAM	Perfect synchronization	-	79.5%
L. Häring [54]	Signalling-assisted modulation classifier	M-QAM	Known CSI, knowledge about total number of loaded bits and coding rate	AWGN	98.5%
L. Häring [55]	Jointly optimizes the bit loading algorithm	M-QAM	Perfect synchronization and knowledge about signalling	AWGN	99%
L. Häring [56]	Influence of imperfect reciprocity	IEEE 802.11a/n	Unknown CSI and knowledge about total number of loaded bits	Rayleigh	100%
C. Husmann [57]	MAP Algorithm	BPSK, QPSK, 16-QAM and 64-QAM	Perfect time and frequency synchronization	AWGN	97.5%
S. Bahrani [58]	Improved Approximated MAP Algorithm, channel prediction method	BPSK, QPSK, 16-QAM 64-QAM and no modulation	Perfect synchronization and unknown CSI	AWGN	98%
M. Karabacak [59]	Adaptive Pilot Based	BPSK, QPSK, 16-QAM and 64-QAM	Perfect synchronization and known CSI	AWGN	99.8%
S. bahrani [60]	Rate adaptive (RA) bit loading algorithm	BPSK, QPSK, 16-QAM and no modulation	Perfect synchronization and unknown CSI	Rayleigh	100%

**Table 4:** Summary of FB approaches for OFDM signals.

Author(s)	Feature(s)	Modulation(s)	Parameter(s)	Channel	Decision-Making Approaches	Average PCC at 20 dB SNR
A. D. Pambudi [61]	Mean, Variance, Skewness, Kurtosis and Moment Order	QPSK,16-QAM and 64-QAM	-	Rayleigh	Threshold based technique	91%
D. Shimbo [62]	Amplitude, Moments and Correlation	16-QAM and 64-QAM	Prior knowledge about CFO	AWGN	Threshold based technique	89%
R. Gupta [63]	Using discrete Fourier transform (DFT) and normalized fourth-order cumulants	BPSK, QPSK, MSK, OQPSK, and 16-QAM	Unknown Signal Parameters, unknown CSI and imperfect synchronization	Rayleigh	Likelihood ratio test	97.5%
J. Zhang [64]	Wavelet transform (WT), Transient characteristics	4-FSK, QPSK, 16-QAM and OFDM	Unknown Signal Parameters	Rayleigh	-	100%
Y. Zhu [65]	Kurtosis coefficient, Power spectral parameter, Energy distribution parameter	2-ASK, 4-ASK, 2-FSK, 4-FSK and OFDM	Unknown symbol rate and carrier frequency	AWGN, FNSF, FSF and Rayleigh	Threshold based technique	97%
Y. Ma [66]	Constellation cluster, number of cluster center	QPSK, 8-QAM, 16-QAM, 32-QAM and 64-QAM	Rotation plane and angle	AWGN	Peak-density clustering algorithm	87.5%
Tomoya [67]	Identification estimation method, Modulation parameters of rotation planes and angles	OFDM, CDMA, a block of QAM and so on	-	AWGN	-	92.5%
J. Chen [68]	Inter-class identification, Higher order cumulants	OFDM, 2-FSK, 4-FSK, 8-FSK, BPSK, QPSK, 8-PSK, 16-QAM, 32-QAM and 64-QAM	Perfect CSI	Rayleigh	Threshold based technique	100%
H. Li [69]	Empirical Distribution Function-Based Gaussian Test	M-QAM	Unknown symbol duration, cyclic prefix duration and number of subcarriers	AWGN	-	95%
Y. Liu [70]	Latent Dirichlet Bayesian network, Gibbs sampling method	QPSK, 8-PSK and 16-QAM	Imperfect CSI and unknown SNR	Flat fading	-	97.5%
Y. Liu [71]	Optimal Bayesian Method, latent Dirichlet model, mean field variation inference	QPSK, 8-PSK, 16-QAM, and 16-PSK	Imperfect CSI and unknown SNR	Flat fading	-	97%
A.K. Pathy [72]	Using DFT and normalized fourth-order and sixth-order cumulants	BPSK, QPSK, MSK, OQPSK, and 16-QAM	Unknown Signal Parameters, unknown CSI and imperfect synchronization	Rayleigh	Likelihood ratio test	97%

**Table 5:** Summary of ML-based classifiers for OFDM signals.

Author(s)	Classifier(s)	Modulation(s)	Parameter(s)	Channel(s)	Average PCC at 20 dB SNR
M.L.D. Wong [73]	Optimize Shannon’s channel capacity, Naive Bayes classifier	BPSK, QPSK, 16-QAM and 64-QAM	Perfect synchronization	AWGN	96.8%
S. E. El-Khamy [74]	Higher order moments and cumulants, Fuzzy *K*-Means and Fuzzy *C*-means	BPSK, QPSK, 16 QAM, and 64 QAM	-	Rayleigh	100%
X. Yuan [75]	Higher-order cumulants, random forest based MC algorithm	QPSK, 16-QAM and 64-QAM	Imperfect time synchronization	Frequency- selective	100%
W. Machid [76]	Least squares (LS) method and iterative closest point (ICP)	BPSK, QPSK, 16-QAM, and 64-QAM	Unknown noise variance and CSI	Flat fading	97.5%
Y. Zhang [77]	High order cumulants, Decision Tree classifier	BPSK, QPSK, GFSK, 16-QAM, 64-QAM and OFDM	Presence of timing offset	Flat fading	99.5%
B. Dehri [78]	Higher order statistics, pattern recognition methods, ANN or SVM, or RFC or KNN	QPSK and 16-QAM	Presence of CFO and Imperfect CSI	Rayleigh	100%
Y. Gu [79]	Peaks in the distribution of amplitude, the variance of the amplitude, the variance of the phase, and the variance of the spectrum, SVM classifier	BPSK, QPSK, 16-QAM, 64-QAM, 256-QAM and GMSK	Unknown CFO	AWGN	100%
J. He [80]	Clustering and Gaussian model	QPSK, 16-QAM, 64-QAM	-	AWGN	100%
L. Gaohui [81]	High order cumulants and bi-spectral envelope peaks, hierarchical iterative SVM classifier model	M-QAM, MFSK and MPSK	Perfect synchronization	Rayleigh	100%

**Table 6:** Summary of DL-based classifiers for OFDM signals.

Author(s)	Classifier(s)	Modulation(s)	Parameter(s)	Channel(s)	Average PCC at 20 dB SNR
R. M. Al-Makhlasawy [82]	Mel Frequency Cepstral Coefficients (MFCCs) and multi-layer feed -forward neural network	QPSK, 8-QAM, 16-QAM, 32-QAM, 64-QAM and 128-QAM	Perfect synchronization	AWGN	100%
Y. Li [83]	Bispectrum and CNN Alexnet model	BPSK, 2-ASK, 2-FSK, 4-FSK, 8-FSK, LFM, and OFDM	-	AWGN	97.5%
S. Hong [84]	CNN with dropout layer	BPSK, QPSK, 8-PSK, 16-QAM and 64-QAM	Perfect synchronization	Rician fading	99%
J. Shi [85]	CNN, ReLU and PReLu activation	BPSK, QPSK, 8-PSK, and 16-QAM	Presence of phase offset and imperfect CSI	AWGN	100%
S. Hong [86]	CNN	BPSK, QPSK, 4-PAM, 8-PSK, and 16-QAM	Perfect synchronization	Rician fading	97.5%
F. Meng [87]	CNN with two step training, Transfer learning	BPSK, QPSK, 8-PSK, 16-PSK, 16-QAM, 32-PSK and 64-QAM	Unknown CFO and unknown SNR	Time invariant and frequency non-selective	100%
D. H. AlNuaimi [88]	GaFP-Net, TF-HMS, MDNC, and IQL	QPSK, BPSK, DPSK, ASK, FSK, 16-QAM, 32-QAM, 64-QAM, and 128-QAM	Unknown CFO	AWGN	86%
Z. Zhang [89]	CNN-LSTM	BPSK, QPSK, 8-PSK, AM-DSB, AM-SSB, CPFSK, GFSK, WBFM, 4-PAM, 16-QAM, and 64-QAM	Presence of CFO and STO	Rayleigh	91%
M.C. Park [90]	IQ and FFT window bank (FWB), CNN-LSTM-based classifier	QPSK, 16-QAM, 32-QAM, and 64-QAM	-	Rayleigh	98.5%
Y. Zhang [91]	Mixed order moment, hybrid grey wolf optimization (HGWO) algorithm, DNN-based classifier	QPSK, 16-QAM, 32-QAM, and 64-QAM	Presence of CFO and STO	Rayleigh	100%
Z. Zhao [92]	AlexNet/GoogLeNet- TL-based classifier	BPSK, QPSK, 8-QAM, 16-QAM, 32-QAM and 64-QAM	-	AWGN	100%
J. Yin [93]	Lightweight CNN (LCNN)-based Shuffle MC, FFT, $l_{2}$ regularization	BPSK, QPSK, 8-PSK, 16-QAM	-	Rician fading	100%
G. Kong [94]	Fourier synchrosqueezing transformation (FSST), Independent component analysis (ICA), hierarchical CNN-based MC	16-QAM, 64-QAM, and 256-QAM	Perfect Synchronization	Rayleigh	90%
Q. Zheng [95]	Spectrum interference-based two-level data augmentation method, deep CNN	BPSK, QPSK, 8-PSK, 16-QAM, 64-QAM, GFSK, CPFSK, 4-PAM, WBFM, AM-SSB, and AM-DSB	-	Rayleigh	89.3%
T. Huynh-The [97]	CNN with integrated attention and residual connections	BPSK, QPSK, 8-PSK, and 16-QAM	Presence of CFO	Rayleigh	88%

## Data Availability

Not applicable.
